# Altered Regional Homogeneity in Patients With Congenital Blindness: A Resting-State Functional Magnetic Resonance Imaging Study

**DOI:** 10.3389/fpsyt.2022.925412

**Published:** 2022-06-22

**Authors:** Jiong-Jiong Hu, Nan Jiang, Jun Chen, Ping Ying, Ming Kang, San-Hua Xu, Jie Zou, Hong Wei, Qian Ling, Yi Shao

**Affiliations:** ^1^Department of Ophthalmology, Zhongshan Hospital Fudan University, Shanghai, China; ^2^Molecular Neuropharmacology Laboratory, School of Optometry and Ophthalmology and Eye Hospital, Wenzhou Medical University, Wenzhou, China; ^3^Jiangxi University of Traditional Chinese Medicine, Nanchang, China; ^4^Department of Ophthalmology, The First Affiliated Hospital of Nanchang University, Nanchang, China

**Keywords:** congenital blindness, regional homogeneity, resting-state fMRI, anxiety, depression

## Abstract

In patients with congenital blindness (CB), the lack of any visual experience may affect brain development resulting in functional, structural, or even psychological changes. Few studies to date have addressed or focused on the synchronicity of regional brain activity in patients with CB. Our study aimed to investigate regional brain activity in patients with CB in a resting state and try to explain the possible causes and effects of any anomalies. Twenty-three CB patients and 23 healthy control (HC) volunteers agreed to undergo resting state functional magnetic resonance imaging (fMRI) scans. After the fMRI data were preprocessed, regional homogeneity (ReHo) analysis was conducted to assess the differences in brain activity synchronicity between the two groups. Receiver operating characteristic (ROC) curve analysis was used to explore whether the brain areas with statistically significant ReHo differences have diagnostic and identification values for CB. All CB patients were also required to complete the Hospital Anxiety and Depression Scale (HADS) to evaluate their anxiety and depression levels. The results showed that in CB patients mean ReHo values were significantly lower than in HCs in the right orbital part of the middle frontal gyrus (MFGorb), bilateral middle occipital gyrus (MOG), and the right dorsolateral superior frontal gyrus (SFGdl), but significantly higher in the left paracentral lobule (PCL), right insula and bilateral thalamus. The ReHo value of MFGorb showed a negative linear correlation with both the anxiety score and the depression score of the HADS. ROC curve analysis revealed that the mean ReHo values which differed significantly between the groups have excellent diagnostic accuracy for CB (especially in the left PCL and right SFGdl regions). Patients with CB show abnormalities of ReHo values in several specific brain regions, suggesting potential regional structural changes, functional reorganization, or even psychological effects in these patients. FMRI ReHo analysis may find use as an objective method to confirm CB for medical or legal purposes.

## Introduction

Congenital blindness (CB) refers to a group of diseases or conditions occurring in the neonatal period or infancy that result in permanent blindness later in life if left untreated (1). In China, the incidence of blindness in children under the age of 5 is <0.10% or about 70000 children in this age range (2). The three most common causes of blindness in Chinese children are cataract, retinal dystrophy, and optic nerve hypoplasia (3). The development of vision is a complicated neural process. Visual stimulation begins at photoreceptors and then travels to the brain's visual centers *via* a series of pathways and connections (4). Normal visual function requires the optics of the eye and the retinal/brain neural networks to function well. Studies have found that visual deprivation before the critical period is likely to cause functional reorganization of the brain (5) with the visual cortex or visual-related area becoming more involved in non-visual information processing such as auditory, tactile, or cognitive tasks (6). In individuals with CB, the visual cortex may process non-visual information through cross-modal plasticity (7, 8). In addition, many studies have reported brain anatomical changes in patients with CB (9). For instance, CB patients may have a thicker occipital cortex than sighted individuals (10) and significantly reduced lateral geniculate nucleus (LGN) volume (11). Therefore, CB may be closely associated with these brain dysfunctions or anatomical abnormalities.

Static functional magnetic resonance imaging (fMRI) is a reliable and informative method to study brain activity in a resting state and has been widely applied in CB patients. The regional homogeneity (ReHo) method has proven a reliable and sensitive resting-state fMRI data analysis method since it was introduced in 2004 (12). Each voxel's Kendall coefficient of concordance is obtained by analyzing the consistency between its blood oxygen level-dependent signals and that of 26 adjacent voxels in a given time period. Higher ReHo value indicates greater consistency. We have applied the ReHo method to evaluate the regional consistency of brain activity in patients with eye disease or conditions including acute eye pain (13), corneal ulcer (14), diabetic optic neuropathy (15), diabetic retinopathy (16), dry eye (17), dysthyroid optic neuropathy (18), late monocular blindness (19), optic neuritis (20), post-ophthalmectomy (21), retinal detachment (22), retinal vein occlusion (23), and strabismus (24, 25). In the present research, we use fMRI and ReHo analysis intending to determine whether spontaneous brain activity is normal in CB, and thus to better understand these patients.

## Subjects and Methods

### Subjects

Twenty-three CB patients (15 males and 8 females, mean age 14.80 ± 2.03 years) were enrolled in the CB group. They were all recruited from the Ophthalmology Department of the First Affiliated Hospital of Nanchang University. Since birth, they had minimal or no visual experience and had no history or objective signs of light perception (response to light or objects). In most of the patients, CB was due to retinopathy of prematurity, but in some cases, the cause was congenital glaucoma, ocular tumor, or unknown. Demographic information and the causes of CB are summarized in Table 1.

**Table 1:** Demographic data of congenital blind individuals.

**ID**	**Sex**	**Age**	**Dx**	**Residual**	**Handness**	**Etiology**
			**month**	**vision**	
CB01	M	14	3	NLP	Right	Retinopathy of pre-maturity
CB02	M	16	2	NLP	Right	Retinopathy of pre-maturity
CB03	M	17	8	NLP	Right	Retinopathy of pre-maturity
CB04	M	11	2	NLP	Right	Retinopathy of pre-maturity
CB05	F	14	1	NLP	Right	Retinopathy of pre-maturity
CB06	M	17	Birth	NLP	Right	Unknown
CB07	M	15	Birth	NLP	Right	Unknown
CB08	F	12	3	NLP	Right	Retinopathy of pre-maturity
CB09	F	16	3–5	NLP	Right	Retinopathy of pre-maturity
CB10	M	17	Birth	NLP	Right	Congenital glaucoma
CB11	F	17	Birth	NLP	Right	Retinopathy of pre-maturity
CB12	M	15	Birth	NLP	Right	Retinopathy of pre-maturity
CB13	M	18	Birth	NLP	Right	Retinopathy of pre-maturity
CB14	M	16	Birth	NLP	Right	Unknown
CB15	M	17	3–5	NLP	Right	Unknown
CB16	F	15	2	NLP	Right	Retinopathy of pre-maturity
CB17	F	15	1	NLP	Right	Retinopathy of pre-maturity
CB18	M	15	4	NLP	Right	Retinopathy of pre-maturity
CB19	M	15	Birth	NLP	Right	Unknown
CB20	F	11	3–4	NLP	Right	Ocular tumors
CB21	M	13	Birth	NLP	Right	Unknown
CB22	F	14	3–5	NLP	Right	Retinopathy of pre-maturity
CB23	M	11	Birth	NLP	Right	Unknown

*Dx months, the age of months of subjects first found as congenital blindness; NLP, no light perception*.

Twenty-three (15 male and 8 female) age-, gender-, and handedness-matched subjects were also enrolled in the healthy control (HC) group with best corrected visual acuity better than 0.8 (decimal notation) with no severe ocular disease history. Subjects from both groups met the following criteria: (i) no systemic disease history and no abnormalities found on brain MRI; (ii) no history of mental illness; (iii) no contraindications to MRI scans.

Our research followed the Declaration of Helsinki and was approved by the Medical Ethics Committee of the First Affiliated Hospital of Nanchang University. Every subject voluntarily participated in this research after understanding the objective of this research and the possible risks.

### MRI Parameters

The magnetic resonance imaging was completed by MAGNETOM Trio 3T (Siemens AG, Munich, Germany). All subjects were required to close their eyes, keep their heads still, stay awake and relax during the scanning. Conventional T1 weighted image (T1WI), conventional T2 weighted image (T2WI), three-dimensional T1-weighted gradient-echo (3D T1W GRE) imaging, and resting-state fMRI data were all collected. The 3D T1W GRE scan parameters were set as repetition time(TR) 1,900 ms, echo time (TE) 2.26 ms, the field of view 250 × 250 mm, flip angle 90°, acquisition matrix 256 × 256, thickness 1.0 mm, gap 0.5 mm, voxel size = 1 × 1 × 1mm^3^. Two hundred and forty functional images were also obtained. The echo-planar imaging (EPI) scan parameters were set as TR 2,000 ms, TE 30 ms, field of view 220 × 220 mm, flip angle 90°, acquisition matrix 64 × 64, thickness 4.0 mm, gap 1.2 mm. The scanning time was 5 and 10 min for each.

### fMRI Data Processing

Raw image data were first filtered by MRIcro software (https://people.cas.sc.edu /rorden/mricro/mricro.html) and then preprocessed by using Statistical Parametric Mapping software (http://www.fil.ion.ucl.ac.uk/spm/) and Data Processing Assistant for Resting-State fMRI v4.0 software (http://rfmri.org/DPARSF). To reduce the influence of the initial magnetic field instability of magnetic resonance, the first 10 scans were removed from the analysis. Slice time correction is used to eliminate scan errors at different timing. And the head motion was corrected to make the scanning results more reliable. Then co-registration and spatial normalization (Montreal Neurological Institute Brain Atlas, resampling voxels = 3 × 3 × 3 mm^3^) were executed to allow the fMRI scanning results for group analysis. To remove the impact of physiological noise and low-frequency drift, band-pass temporal filtering and linear detrending were performed. After ReHo analysis, we applied spatial smoothing by using the Gaussian kernel (full width at half maximum, FWHM = 6 mm) to improve the signal-to-noise ratio.

### Statistical Analysis

Resting State fMRI Data Analysis Toolkit software (REST, http://www.restfmri.net /forum/) was run for getting the ReHo value of every voxel by calculating the Kendall coefficient of concordance (KCC) of adjacent 26 voxels. KCC's calculation is shown as follow:


$$\begin{array}{l} W=12\frac{\sum_{i=1}^{n}{\left(R_{i}-\bar{R}\right)}^{2}}{K^{2}\left(n^{3}-n\right)}\; \end{array}$$

where W represents the KCC among given voxels; K represents the number of neighbors, which is 27 in this study; n represents the number of total time points; R_i_ represents the sum of the rank of signals of a certain voxel and its neighbors at time point i; $\bar{R}$ represents the average of R_1_ to R_n_, which means the overall mean rank of all voxels in the cluster throughout the total time series. And ReHo value= standardized ReHo (Z-score) = (KCC of this voxel–average KCC of total voxels)/standard deviation of KCC of total voxels (26). The ReHo value represents the consistency of the BOLD signal between a certain voxel and the surrounding voxels within a period of time and reflects the consistency of the brain activities in the specific area. The higher the ReHo value, the stronger consistency of the local brain activities have. An independent samples *t*-test was carried out by REST Software to detect the significant ReHo differences between the two groups. The statistical threshold was set at the voxel level with *P* < 0.05 and multiple comparison was conducted with false discovery rate (FDR) corrected and cluster size > 50 voxels. ROC curves were used to identify the CB patients by certain brain region ReHo values. The area under the ROC curve (AUC) was computed to evaluate the diagnostic ability. In addition, all the CB patients were provided with vocal version of the Hospital Anxiety and Depression Scale (HADS) (27), and the results were collected. We then use the anxiety score and depression score obtained in the HADS to assess the CB patient's anxiety and depression level (Normal: 0 ~ 7, Borderline: 8 ~ 10, Abnormal: 11 ~ 21). The onset and the duration of blindness in the CB group were also recorded. Pearson correlation analysis was performed by GraphPad Prism 9 software (https://www.graphpad.com/scientific-software/prism/) to evaluate whether there is a linear correlation between the ReHo value in right MFGorb and the anxiety score/depression score and whether there is a linear relationship between duration of blindness and ReHo values of significantly different brain regions.

## Result

### Demographic Information

Basic information about the two groups is shown in Table 2. There is no difference between the sex ratio, age, and handedness.

**Table 2:** Clinical characteristics of participants in this study.

**Condition**	**CB (mean ±SD)**	**HCs (mean ±SD)**	***T*-value**	***P*-value**
Male/female	15/8	15/8	N/A	>0.99
Age (years)	14.8 ± 2.03	14.70 ± 1.97	0.87	0.88
Handedness	23R	23R	N/A	>0.99
Duration of CB (years)	14.62 ± 2.10	N/A	N/A	N/A

*CB, Congenital blindness; HCs, Healthy controls; SD standard deviation; N/A, not applicable*.

### Regional Homogeneity Differences

**Figure 1:**
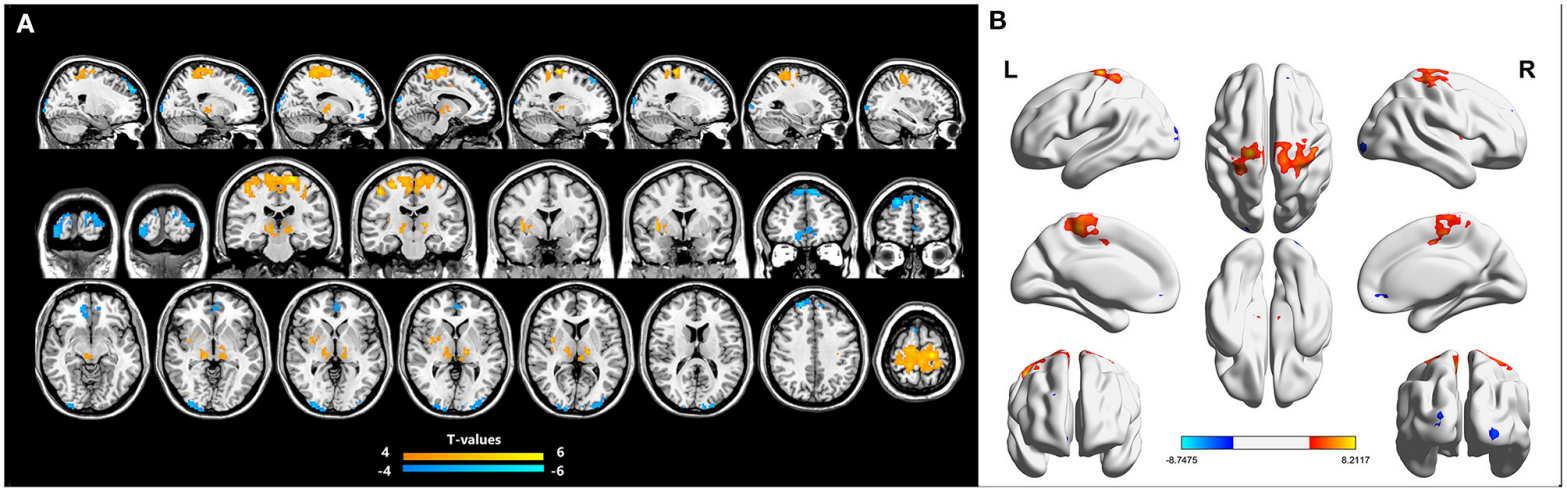
Significantly different ReHo brain areas in patients with congenital blindness. Blue regions (RMFGorb, bilateral MOG, RSFGdl) indicate lower ReHo values, while red regions (the bilateral thalamus, the right insula, and the LPCL) represent higher ReHo values (single voxel *P* < 0.05, AlphaSim corrected; cluster size > 50). The map of the brain regions with statistical differences between two groups **(A)** and three-dimensional distributions **(B)**. ReHo, regional homogeneity; RMFGorb, the right orbital part of middle frontal gyrus; MOG, the middle occipital gyrus; RSFGdl, the right dorsolateral superior frontal gyrus; LPCL, the left paracentral lobule.

**Figure 2:**
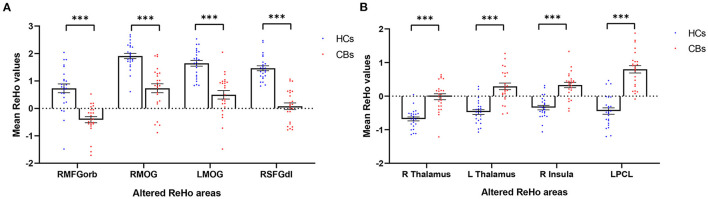
Mean ReHo values (Z-score) of significantly altered brain areas between the CB and HC groups. **(A)** Compared to HCs, The mean ReHo values of the following regions were decreased in CB patients: RMFGorb (*t* = −5.62), the RMOG (*t* = −5.96), LMOG (*t* = −5.94), and the RSFGdl (*t* = −8.75). **(B)** Compared to HCs, the mean ReHo values of the following regions were increased in CB patients: the R Thalamus (*t* = 5.98), the L Thalamus (*t* = 6.23), the R insula (*t* = 6.2), the LPCL (*t* = 8.21). ReHo, regional homogeneity; CB, congenital blindness; HC, healthy control; R, right; L, left; RMFGorb, right orbital part of middle frontal gyrus; RMOG, right middle occipital gyrus; LMOG, left middle occipital gyrus; RSFGdl, right dorsolateral superior frontal gyrus; LPCL, left paracentral lobule. ***Represents the significant differences in ReHo value between the two groups (*p* < 0.001).

Figures 1A,B show the brain regions in which ReHo differences were significantly different between the two groups. For CB patients, the mean ReHo values (Z-score) were significantly lower than controls in the right orbital part of the middle frontal gyrus (RMFGorb), bilateral middle occipital gyrus (MOG), and right dorsolateral superior frontal gyrus (RSFGdl), but higher than controls in the left paracentral lobule (LPCL), right insula and bilateral thalamus (Figures 1A,B, Table 3). The mean standardized ReHo values in these regions are shown in Figure 2.

**Table 3:** Brain areas with significantly different ReHo values between groups.

**ReHo**	**Side**	**Brain**	**Peak**	***T*-value**	**MNI coordinates**		
**difference**		**areas**	**voxels**	
					**X**	**Y**	**Z**
CB < HC	Right	Orbital part of MFG	104	−5.62	12	45	−9
	Right	Middle occipital gyrus	112	−5.96	30	−96	0
	Left	Middle occipital gyrus	81	−5.94	−21	−99	15
	Right	Dorsolateral SFG	352	−8.75	21	54	39
CB > HC	Right	Thalamus	80	5.98	12	−24	−3
	Left	Thalamus	68	6.03	−15	−18	6
	Right	Insula	54	6.2	36	0	0
	Left	Paracentral lobule	1,547	8.21	−18	−24	69

*The correction level was set voxel-wisely P < 0.05 FDR corrected, and cluster size > 50 voxels for multiple comparison. ReHo, regional homogeneity; CB, congenital blindness; HC, healthy control; BA, Brodmann area; MNI, Montreal neurological institute; MFG, middle frontal gyrus; SFG, superior frontal gyrus*.

### ROC Curve Analysis

**Figure 3:**
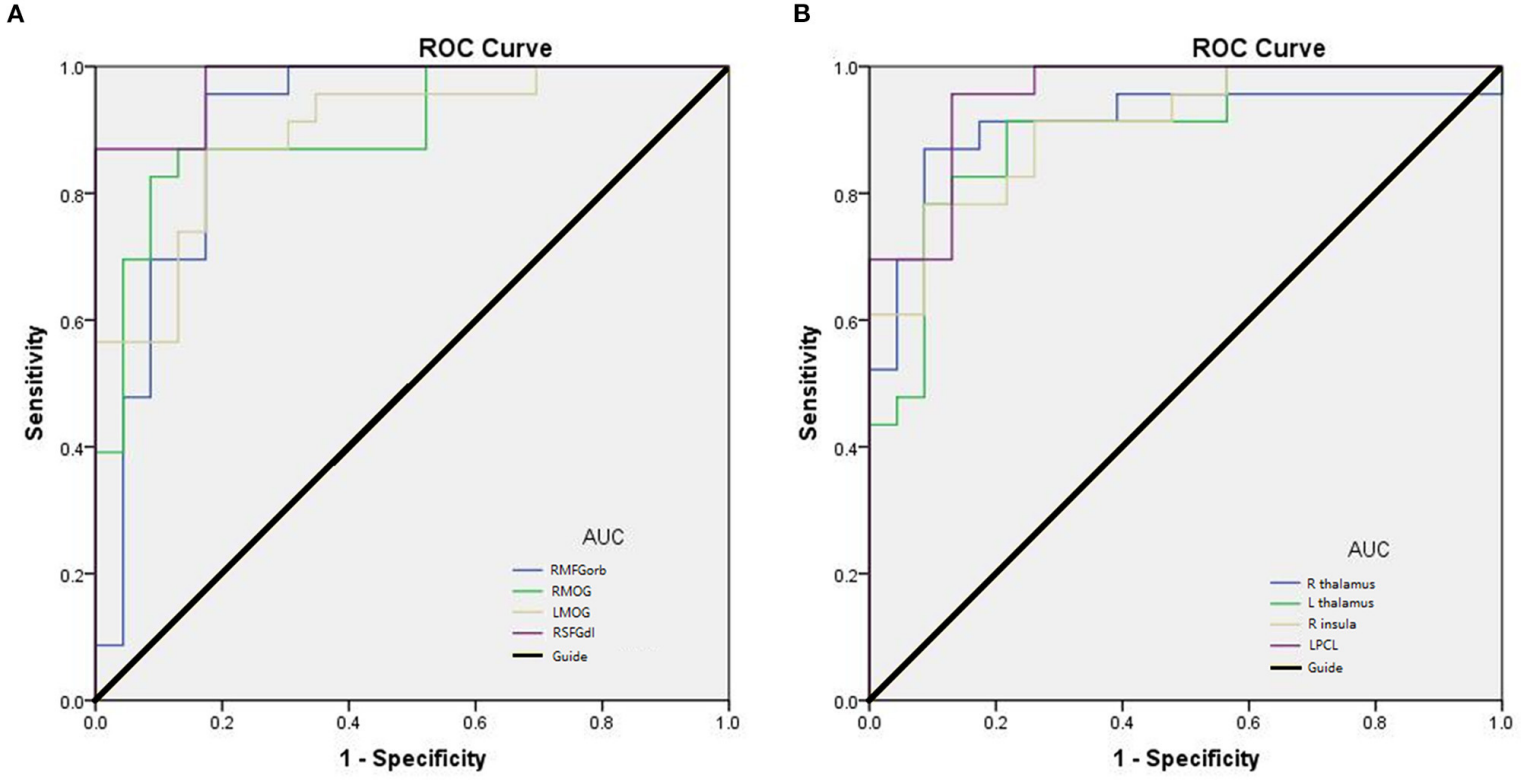
Receiver operating characteristic (ROC) curve analysis of mean ReHo in corresponding brain regions. **(A)** The AUC were 0.905 [*p* < 0.0001; 95% CI (0.810–1.000)] for the RMFGorb; RMOG 0.902, [*p* < 0.0001; 95% CI (0.812–0.992)]; LMOG 0.896, [*p* < 0.0001; 95% CI (0.806–0.986)]; RSFGdl 0.977, [*p* < 0.0001; 95% CI (0.944–1.000)]. **(B)** The AUC were 0.909 [*p* < 0.0001; 95% CI (0.821–1.000)] for R thalamus; L thalamus 0.898, [*p* < 0.0001; 95% CI (0.807–0.989)]; R insula 0.907, [*p* < 0.0001; 95% CI (0.825–0.990)]; LPCL 0.955, [*p* < 0.0001; 95% CI (0.902–1.000)]. AUC area under the curve; CI confidence interval; R, right; L, left; RMFGorb, right orbital part of middle frontal gyrus; RMOG, right middle occipital gyrus; LMOG, left middle occipital gyrus; RSFGdl, right dorsolateral superior frontal gyrus; LPCL, left paracentral lobule.

We hypothesized that the mean ReHo values of certain brain areas may be an effective marker for CB. To test this hypothesis, we generated receiver operating characteristic (ROC) curves to analyze the responses in specific brain areas with significant ReHo differences between groups. The regions with lower area under the curve (AUC) for CB than for HC are as follows: RMFGorb, AUC 0.905 [*p* < 0.0001; 95% CI (0.810–1.000)]; RightMOG, 0.902 [*p* < 0.0001; 95% CI (0.812–0.992)]; LeftMOG, 0.896 [*p* < 0.0001; 95% CI (0.806–0.986)]; and RSFGdl, 0.977 [*p* < 0.0001; 95% CI (0.944–1.000); Figure 3A]. Regions in which AUC was higher in CB than in HC were: LPCL, 0.955 [*p* < 0.0001; 95% CI (0.902–1.000)]; Right Thalamus, 0.909 [*p* < 0.0001; 95% CI (0.821–1.000)]; Left Thalamus, 0.898 [*p* < 0.0001; 95% CI (0.807–0.989)]; and Right Insula 0.907 [*p* < 0.0001; 95% CI (0.825–0.990); Figure 3B].

### Correlation Analysis

**Figure 4:**
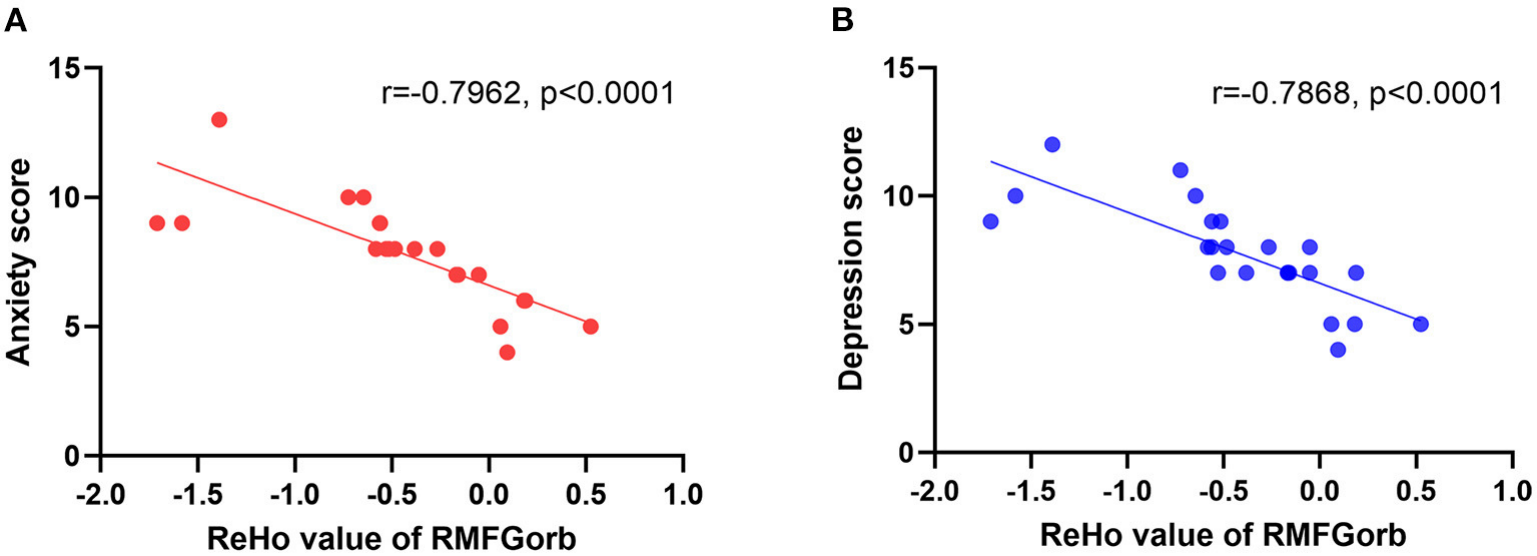
The linear relationship between the ReHo value of the RMGForb and the anxiety score/depression score. **(A)** The anxiety score obtained from the HADS was negatively correlated with the ReHo value of RMFGorb, *r* = −0.7962, *p* < 0.0001; **(B)** The depression score obtained from the HADS was also negatively correlated with the ReHo value of RMFGorb, *r* = −0.7868, *p* < 0.0001. ReHo, regional homogeneity; RMFGorb, right orbit part of middle frontal gyrus; HADS, Hospital Anxiety and Depression Scale.

Pearson's correlation was used to analyze the relationship between the ReHo value of RMFGorb and the anxiety and depression scores obtained from the HADS, and between the altered brain regions and the duration of CB. It is worth noting that among the 23 patients with CB, only 9 had normal HADS scores; one patient was in a state of depression and anxiety, one was in a state of depression and borderline anxiety, nine were in a state of borderline depression and borderline anxiety, two had borderline anxiety only, and one had borderline depression only. The ReHo value of RMFGorb was significantly negatively correlated with both the HADS anxiety score (Figure 4A) and depression score (Figure 4B), but no linear relationship was found between the duration of blindness and the mean ReHo values in the altered brain areas (Table 4).

**Table 4:** Pearson correlation analysis between the ReHo values in the altered brain areas and the duration of blindness.

**Brain**	**ReHo value**	**Duration (years)**	***R*-value**	***P*-value**
**regions**	**(mean ±SD)**	**(mean ±SD)**	
RMFGorb	−0.413 ± 0.543	14.623 ± 2.098	0.05043	0.8192
RMOG	0.732 ± 0.798		0.06588	0.7652
LMOG	0.495 ± 0.747		−0.1096	0.6185
RSFGdl	0.078 ± 0.595		0.06702	0.7613
R thalamus	−0.018 ± 0.433		−0.1687	0.4416
L thalamus	0.291 ± 0.485		−0.2172	0.3194
R insula	0.330 ± 0.393		−0.1279	0.5608
LPCL	0.799 ± 0.539		0.1392	0.5265

*ReHo, regional homogeneity; SD, standard deviation*.

## Discussion

Functional MRI may be used to measure cerebral activity and simultaneously determine the active location. This provides an effective and reliable means by which to understand the brain activity anomalies in cognition-related disorders or diseases. Resting-state fMRI does not require patients to perform specific tasks during scans, so it can reduce the influence of motor neural activity which occurs in a task-based fMRI scan. In addition, ReHo analysis has been used to reveal abnormal brain activity in ophthalmology (Table 5).

**Table 5:** Application of ReHo in ophthalmological diseases.

**Diseases**	**Author**	**Year**	**References**
Glaucoma	Song et al.	2014	(28)
Diabetic retinopathy	Cui et al.	2014	(29)
Optic neuritis	Shao et al.	2015	(20)
Open-globe injury	Huang et al.	2016	(30)
Late monocular blindness	Huang et al.	2017	(19)
Retinal detachment	Huang et al.	2017	(22)
Acute eye pain	Tang et al.	2018	(13)
Strabismus and amblyopia	Shao et al.	2019	(25)
Corneal ulcer	Xu et al.	2019	(14)
Retinal vein occlusion	Wen et al.	2019	(23)
Dysthyroid optic neuropathy	Jiang et al.	2021	(18)
Dry eye	Yu et al.	2021	(17)

**Figure 5:**
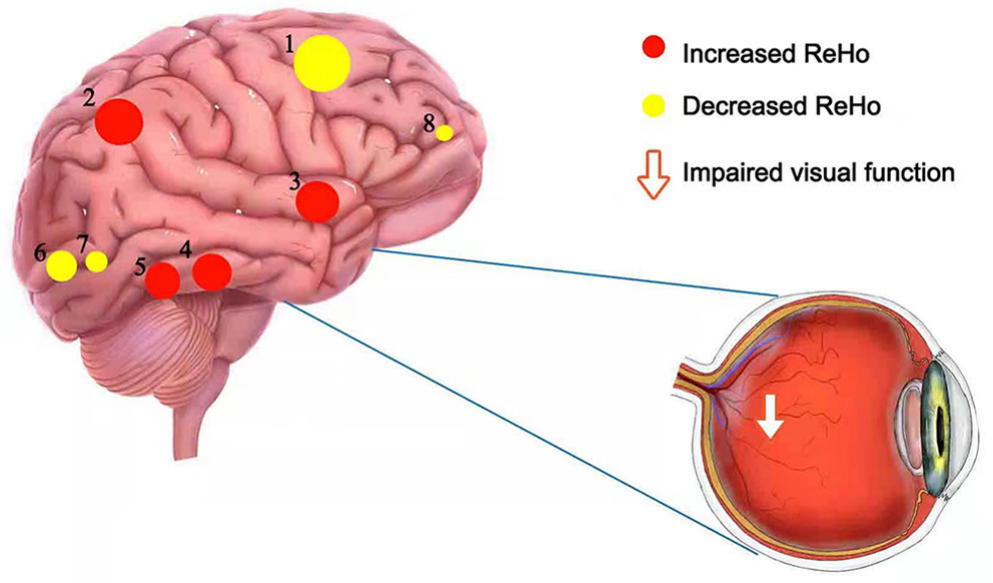
The schematic brain region map of CB patients with significant ReHo differences compared to HC. The ReHo values of CB patients were reduced to varying degrees compared with HCs in the following regions: 8-the right orbital part of the middle frontal gyrus (BA 32, *t* = −5.62), 6-the right middle occipital gyrus (BA 18, *t* = −5.96), 7-the left middle occipital gyrus (BA 18, *t* = −5.94), 1-the right dorsolateral superior frontal gyrus (BA 8, *t* = −8.75). Instead, the ReHo values of CB patients in the following regions were increased compared with HCs: 5-the right thalamus (*t* = 5.98), 4-the left thalamus (*t* = 6.23), 3-the right insula (*t* = 6.2), 2-the left paracentral lobule (BA 3, *t* = 8.21). CB, congenital blindness; HC, healthy control; ReHo, regional homogeneity; BA, Brodmann's area.

In contrast to the HCs, patients in the CB group showed significantly decreased mean ReHo values in the RMFGorb, bilateral MOG, and right posterior dorsolateral part of superior frontal gyrus (RSFGdl_p) (Figure 5).

**Figure 6:**
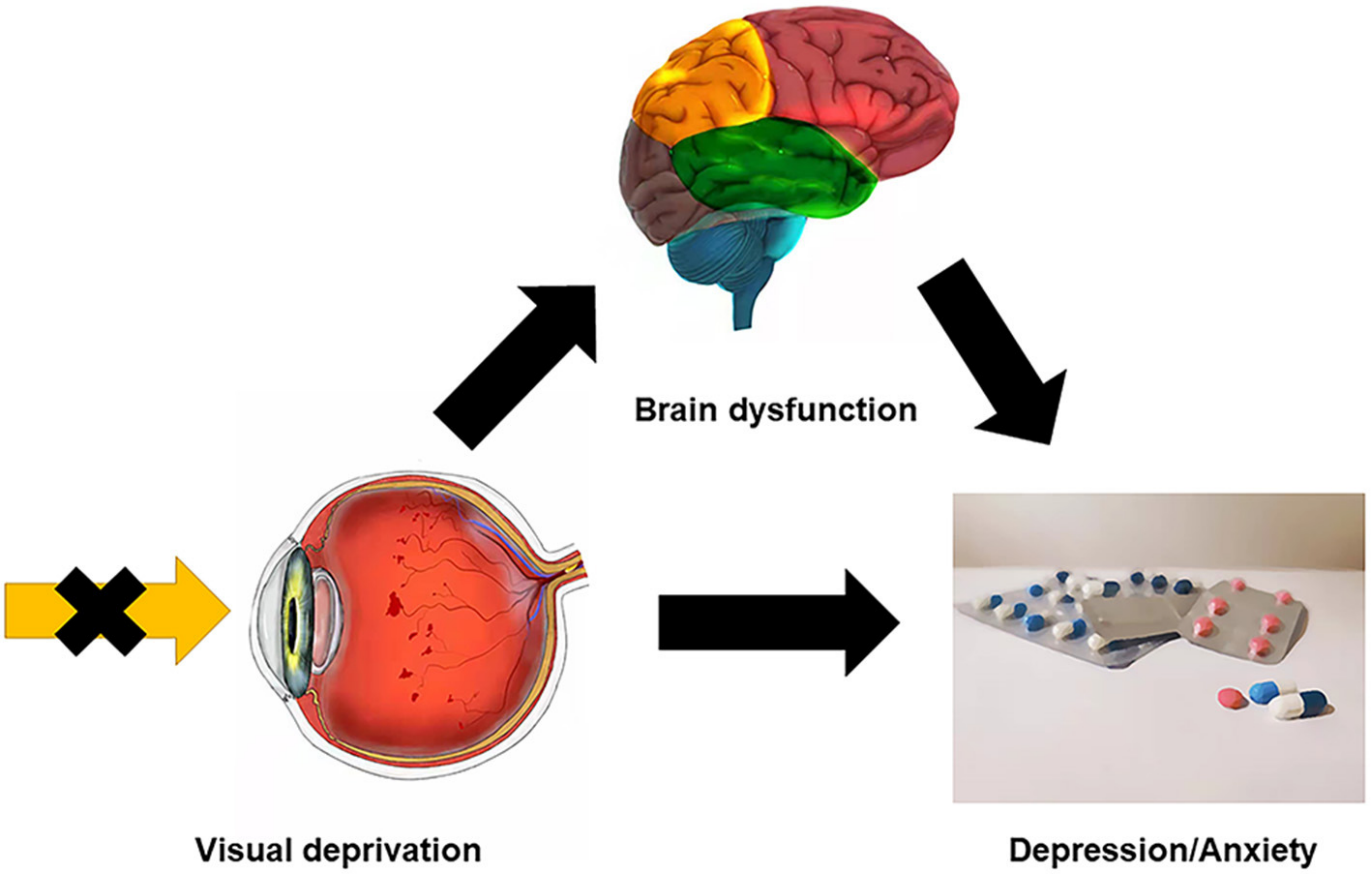
Relationship between clinical manifestation, brain dysfunction, and psychological conditions of CB patients. CB patients could get certain brain region dysfunction because of visual deprivation, for example, the ReHo values were significantly decreased at MFG in CB patients, which could be relevant to anxiety and depression. And the visual disability itself may bring physiological or psychosocial problems, which make CB patients more susceptible to psychological disorders. CB, congenital blindness; MFG, middle frontal gyrus; ReHo, regional homogeneity.

The middle frontal gyrus (MFG) is located in the middle of the frontal lobe. The dorsolateral prefrontal cortex lies in the MFG which may be related to depression (31). The cortical regions associated with visual fixation of peripheral targets are known as the frontal eye fields (FEF) and lie in a region around the junction of the MFG and the precentral gyrus. The FEF is thought to be associated with the control of eye movements and visual attention since unilateral FEF impairment is associated with both eyes deviating to the ipsilateral side. CB patients are not able to consciously move their eyes and lack the vestibulo-ocular reflex while eye movement is practically normal in those with acquired blindness (32, 33). Since visual input is absent from birth in CB patients, their voluntary eye movements may be compromised. The MFG has also been found to be involved in cognitive function and emotion processing. Previous studies indicate that the right MFG may play a part in contingency awareness (34) and reorienting attention (35), that right MFG gray matter volume is reduced in late-life depression and remitting depression patients (31, 36), and that activity of the right orbital part of MFG is reduced in patients with depression and anxiety (37). It is known that visual impairment and difficult living conditions will have a notable psychological impact on CB patients. Anomalies may occur in the brain regions associated with emotional processing, and the fact that 14 of 23 CB patients in our study showed borderline or abnormal states of anxiety or depression is consistent with this possibility. The abnormal changes at RMFGorb may help us to detect anxiety or depression in CB patients earlier. Other studies have also found that CB patients are indeed prone to suffer from depression and anxiety (38–40). We found a significantly decreased mean ReHo value at the RMFGorb in CB patients compared with HCs and the ReHo value of RMFGorb in CB patients was negatively correlated with their anxiety and depression scores. These findings may be related to the impaired voluntary eye movements in CB patients due to their visual deprivation and may be associated with the psychiatric changes such as depression and anxiety that occur in CB patients (Figure 6).

The occipital lobe is located at the posterior part of the cerebrum and is known as the vision and visual processing center (41). The primary visual cortex (also known as the striate cortex, V1) is at the medial and posterior part of the occipital lobe within the calcarine sulcus in each hemisphere. Goodale and Milner first established the classic hypothesis of two visual pathways (42). They believed the primary visual cortex projects information to two pathways, namely the dorsal/localization or “where” stream, mainly processing motion or stereoscopic depth visual information, and the ventral/identification pathway or “what” stream, mainly dealing with colors, shapes and other detailed visual information. However, some researchers have found that the two pathways may not be so clearly distinguished (43, 44). Congenitally blind patients lack visual experience, which suggests that their visual pathway does not develop normally. Previous studies have found that people with early-onset blindness may have brain anatomical changes such as decreased white matter volume in part of the visual pathway and decreased gray matter volume in the visual cortex (45). Despite the anatomical changes, less functional connectivity within the occipital lobe has also been found in early-onset blindness (46). Three gyri (the superior, the middle, and the inferior occipital gyrus) are located along the lateral portion of the occipital lobe (47). Studies have shown that the occipital cortex of people with CB is engaged in other forms of sensory processing, since the occipital cortices, the lingual-gyrus (Brodmann area 18), parts of the cuneus, and the MOG were activated more in early blindness than in sighted control subjects undertaking auditory and tactile tasks (48, 49). The right MOG is part of the dorsal/localization stream and shows increased activity during spatial tactile and auditory tasks in people with early-onset blindness. However, the bilateral MOG showed significantly decreased ReHo values in CB compared to HCs in our resting-state fMRI test, implying that spatial tactile and auditory task-related areas may be inhibited in the resting state.

The superior frontal gyrus (SFG), also known as the marginal gyrus, is located in the superior part of the prefrontal cortex (50) and is involved in higher cognitive functions such as working memory (51). The audio-spatial working memory of CB patients is compromised since visual experience supports spatial memory (52). Significantly reduced ReHo values have been demonstrated in the right dorsolateral part of Brodmann area 8, located at the posterior dorsolateral part of SFG (SFGdl_p), in CB patients (53). This area includes the FEF, known to be related to voluntary eye movements, visual search, and attention control (54, 55). The right and left FEF have slightly different functions, the right FEF playing a role in signal integration (56). The SFGdl_p also has a connection with brain regions that are associated with cognitive control, such as the posterior parietal cortex and dorsolateral prefrontal cortex (57). While FEF may be related to stronger endogenous orienting of non-visual attention (such as verbal cues) in CB patients (58), ReHo is decreased in the right SFGdl_p of CB patients compared with HCs.

However, the mean ReHo values of the CB group in the left paracentral lobule (PCL), right insula, and bilateral thalamus were significantly higher compared with those in HCs (Figure 5).

The PCL is on the medial side of each cerebral hemisphere and is responsible for controlling motor innervations and processing sensory information of the contralateral lower limbs. The left side upper postcentral gyrus (Brodmann area 3) is the precise area with increased ReHo value. The postcentral gyrus is located at the lateral parietal lobe and is also known as the primary somatosensory cortex (59). All of the CB patients and HCs in the present study were right-handed. Increased ReHo of the postcentral gyrus contralateral to the dominant hand in CB patients indicates that they may have more sensitive tactile perception than controls. Several behavioral studies also support the concept of superior tactile abilities in early-blind persons (60, 61). One previous study also found increased activity in the postcentral gyrus in CB subjects compared with sighted individuals on a tactile task (62), consistent with our suggestion.

The insular cortex folds deep within the lateral sulcus in each hemisphere of the brain. The insulae are generally thought to be involved in multimodal sensory processing, taste, self-awareness, motor control, homeostasis, auditory perception, salience, emotions, and other functions. People with CB are thought to be highly sensitive to salient non-visual stimuli. Enhanced intra- and inter-network connectivity such as salience network and frontoparietal network in CB may optimize their abilities to use non-visual stimuli and their attention control, which may help them to adapt to their environment (63). The anterior insula (AI) is believed to be part of the salience network that detects and facilitates higher cognitive processing of the most relevant stimuli in a multitude of forms of sensory information. The functional connectivity between the dorsal AI and the dorsal/localization (where) stream and between the ventral AI and the ventral/identification (what) stream is enhanced in CB (64). This may support our finding that the bilateral insulae are more activated in CB in the resting state. The AI cortex is also believed to be engaged in emotional cognition (empathy), interoceptive awareness (hunger, thirst) and may play a role in conveying this information to consciousness (65). fMRI results show that the AI is correlated with the ability to feel one's own heartbeat and emotion elicitation (66). Increased activity and gray matter volume of the right AI in normal subjects are correlated with higher accuracy of interoceptive sense (measuring own heartbeat). In addition, more negative feelings (higher scores on the questionnaire of the Hamilton Anxiety Scale) are correlated with their interoceptive task accuracy (67). Whether these abilities in CB people are superior remains to be studied in the future. In our study, the increased ReHo at the right insula in CB patients may indicate that they have better interoceptive awareness during the resting state compared to sighted individuals.

The thalamus comprises a pair of large oval gray matter nuclei located in the dorsal part of the diencephalon, just beside the third ventricle. The left and right thalami are connected by a gray matter mass. The thalamus plays roles ranging from sensation relay, regulation of memory and emotion, consciousness, and others (68). The nuclei in the thalamus are separated by a Y-shaped white matter plate into anterior, lateral, and medial groups. Various sensory pathways (except olfactory) replace neurons at the thalamus and then project to the cerebral cortex. For instance, the LGN (part of the thalamus) is the most important relay station in the visual pathway. The retinal ganglion cell gathers visual information from the retina and projects it to the LGN which then projects fibers to the primary visual cortex (area V1). Notably, the LGN not only receives neural projections from the retina, but also has extensive interconnections with the thalamus, brainstem, and visual cortex. Brain functional and structural changes occur after early visual deprivation. One study found that the mean volumes of structures along the visual pathway (including LGN) were reduced by 50% in CB patients (69). Although the LGN may have impaired function and reduced size in CB, volumes of other relay nuclei such as the medial geniculate nucleus (MGN), ventrolateral nucleus (VL), and ventral posterior nucleus (VP) were not affected (70). In addition, studies have found that tactile stimulation in people with CB can stimulate occipital cortical activities. This phenomenon is most likely accomplished by the thalamocortical pathway (71). In animal studies, the deprivation of hamsters' visual systems at birth can lead to retinal projections to the auditory thalamus (72) and the primary visual cortices of enucleated opossums receive projections from nuclei other than LGN, including MGN (auditory), VP (sensation), VL (motor), and anterior nuclear groups (alertness, learning) (73). Human studies have shown that in CB patients the occipital cortex is responsive to auditory stimuli and the superior colliculus is recruited by the auditory system (74). The increased ReHo values of the bilateral thalamus in CB patients probably reflect the structural and functional variations due to early visual deprivation.

Pearson correlation analysis demonstrated no linear correlation between the duration of CB and the mean ReHo values in the significantly altered brain regions.

ROC analysis in our study demonstrates the potential diagnostic value of mean ReHo values (Z-score) of the brain regions found to differ between CB patients and HCs (Figure 3). The AUC of each of these regions was >0.85, indicating effective accuracy in the identification of CB. The AUC of RSFGdl and LPCL was as high as 0.977 and 0.955, respectively. However, these CB patients had been blind for some years (mean age 14.80 ± 2.03 years, with minimum 10.8 years old and maximum 17.8 years old), and it remains unclear whether these positive results hold in newborn blind patients. But ReHo analysis do have the potential to be used for legal or medical proof of CB grown-ups since it is an objective examination.

Patients with CB in our study can only be prevented from the cause at early stages. For these patients who have been CB for years, there is currently no good way to restore sight. Future treatments for patients with CB, such as the use of artificial prosthesis (75), may reverse the abnormalities in these altered brain regions. And the ReHo analysis could help in tracking the treatment effect and assess the function restoration.

## Conclusion

Our experimental results found that patients with CB can have regional abnormal brain activities at a resting state. These abnormal neural activities are likely to be associated with the potential regional structural changes, functional reorganization, and even psychological impact on CB patients (Table 6). These abnormal brain regions suggest the possible location of primary or secondary brain damage in CB, which may help to study the pathogenesis and development of CB in the future. ReHo analysis can record changes of regional brain activities non-invasively. It is of great significance use for clinical follow-up and research, and has great application value in the clinic to discover abnormalities related to brain function. Moreover, our ROC result shows ReHo analysis has the potential to be a safe and effective approach to identify CB for medical or legal purpose. It can also be used in diagnosing of patients with CB with complex causes or for whom vision examination is not available. However, our study does have some limitations. Firstly, the sample size of our study was relatively small. Individual differences may have an impact on our result, so a larger population study is needed to prove our findings. In addition, the 15-min scanning time may result in small unavoidable movements which may undermine the accuracy of our analysis. Also, because some of the CB patients didn't have intact medical records, their histories are based on the subjective description of their parents which might be not accurate. In the future, we will minimize the possible influencing factors to obtain more reliable results.

**Table 6:** Altered brain regions and their potential impact.

**Brain regions (ReHo difference)**	**Brain function**	**Possible impact**
RMFGorb (CBs < HCs)	Eye movements, cognitive functions, emotional processing, attention	Impaired voluntary eye movements, depression/anxiety, cognitive activity disorder
Bilateral MOG (CBs < HCs)	Visual related, spacial tactile, & auditory task	Impaired visual function, object recognition
RSGFdl (CBs < HCs)	Visuospatial attention, eye movements, awareness	Impaired voluntary eye movements, awareness
LPCL (CBs > HCs)	Motor & sensory innervations	Superior tactile skills
Right insula (CBs > HCs)	Multimodal sensory processing, cognitive-emotional process, salience, taste, self-awareness	Better interoceptive awareness
Bilateral thalamus (CBs > HCs)	Relaying sensory/motor signals, regulations of consciousness and alertness arousal, attention, motivation	Reorganization of the sensory and motor systems

*RMFGorb, the right orbital part of middle frontal gyrus; MOG, middle occipital gyrus; RSFGdl, the right dorsolateral superior frontal gyrus; LPCL, the left paracentral lobule*.

## Data Availability Statement

The datasets used and/or analyzed during the current study are available from the corresponding author on reasonable request.

## Ethics Statement

The studies involving human participants were reviewed and approved by Medical Ethics Committee of the First Affiliated Hospital of Nanchang University. The patients/participants provided their written informed consent to participate in this study.

## Author Contributions

J-JH and NJ were major contributors, both conceived and designed the experiments, analyzed the data, wrote, and revised the manuscript. JC, PY, MK, and S-HX recruited the patients and healthy controls for the study, performed the MRI experiments, and helped finish the questionnaires. JZ, HW, and QL collected and treated the data. YS designed the study and offered financial support. All authors read and approved the final manuscript.

## Conflict of Interest

The authors declare that the research was conducted in the absence of any commercial or financial relationships that could be construed as a potential conflict of interest.

## Publisher's Note

All claims expressed in this article are solely those of the authors and do not necessarily represent those of their affiliated organizations, or those of the publisher, the editors and the reviewers. Any product that may be evaluated in this article, or claim that may be made by its manufacturer, is not guaranteed or endorsed by the publisher.
